# Effect of fluid elasticity on the emergence of oscillations in an active elastic filament

**DOI:** 10.1098/rsif.2024.0046

**Published:** 2024-05-29

**Authors:** Kathryn G. Link, Robert D. Guy, Becca Thomases, Paulo E. Arratia

**Affiliations:** ^1^ Department of Mathematics, University of California, Davis, CA 95616, USA; ^2^ Department of Mathematical Sciences, Smith College, Northampton, MA 01063, USA; ^3^ Department of Mechanical Engineering and Applied Mechanics, University of Pennsylvania, Philadelphia, PA 19104, USA

**Keywords:** viscoelastic fluid, flagella, dynamic buckling instability, follower force

## Abstract

Many microorganisms propel themselves through complex media by deforming their flagella. The beat is thought to emerge from interactions between forces of the surrounding fluid, the passive elastic response from deformations of the flagellum and active forces from internal molecular motors. The beat varies in response to changes in the fluid rheology, including elasticity, but there are limited data on how systematic changes in elasticity alter the beat. This work analyses a related problem with fixed-strength driving force: the emergence of beating of an elastic planar filament driven by a follower force at the tip of a viscoelastic fluid. This analysis examines how the onset of oscillations depends on the strength of the force and viscoelastic parameters. Compared to a Newtonian fluid, it takes more force to induce the instability in viscoelastic fluids, and the frequency of the oscillation is higher. The linear analysis predicts that the frequency increases with the fluid relaxation time. Using numerical simulations, the model predictions are compared with experimental data on frequency changes in the bi-flagellated alga *Chlamydomonas reinhardtii*. The model shows the same trends in response to changes in both fluid viscosity and Deborah number and thus provides a possible mechanistic understanding of the experimental observations.

## 1. Introduction

Many microorganisms, such as sperm, propel themselves through complex media by deforming their flagella. It has long been observed that the rheology of the surrounding fluid alters the shape and frequency of the flagellum beat of mammalian sperm [1–5], of sea urchin and related marine animal sperm [6,7] and of the bi-flagellated alga *Chlamydomonas reinhardtii* [8,9]. In addition to beat changes, fluid viscosity has been shown to affect the coordination in arrays of cilia that drive cell locomotion [10] and transport mucus [11].

Human sperm and sperm from marine invertebrates exhibit different gait changes in response to high-viscosity environments [5]. It has been hypothesized that the gait changes in mammalian sperm are important for fertilization because they must swim through viscoelastic mucus [2,5]. There have been multiple observations of sperm gaits in mucus and other viscoelastic fluids [1–4], but there has been no systematic documentation of how gradual changes in fluid elasticity affect the gait. Changes in the beat frequency, shape and swimming speed of *C. reinhardtii* in response to both viscous and elastic properties of the surrounding fluid were recently documented [8]. It was observed that the beat frequency was enhanced by fluid elasticity, and the frequency changed non-monotonically with fluid viscosity in viscoelastic fluids.

Fluid elasticity clearly influences the flagellum beat, but the physical mechanism for how fluid rheology shapes the beat is not known. There have been theoretical studies of how fluid elasticity affects the gait for prescribed active motor forces [12,13]. These studies considered the active forces as a travelling wave with a given frequency, and they examined how the fluid rheology affected the resulting shapes. This approach was able to explain the qualitative shape change observed in some sperm species in viscoelastic fluids [3], and the increased amplitude of the beat, and thus increased swimming speed, in artificial swimmers with flexible tails [14]. However, because the frequency of the active forces was prescribed, this approach cannot be used to understand how the beat frequency changes with fluid rheology as observed in [8].

The flagellum beat is powered by dynein motors that form crossbridges between the microtubule doublets that make up the axoneme. These motors generate active shear forces between adjacent doublets, which through interactions with other passive forces and constraints at the base, lead to bending [15]. It is not understood how the motors along the flagellum are coordinated spatially and temporally to produce the observed waves of bending. There are different hypotheses about how mechanical feedback on motor activity from deformations of the flagellum leads to emergent coordination of the whole system. Some of the leading feedback mechanisms that have been explored are that the motors respond to changes in curvature [16–18], tangential deformations (i.e. sliding control) [19,20] or normal forces (i.e. ‘geometric clutch’) [21,22]. All of these mechanisms have been explored thoroughly in models, and they are all capable of producing emergent waves in the flagellum. Analysis of the bifurcation structure of the three models cast doubt on the sliding-control mechanism [23], though sliding control was capable of matching experimental data on bull sperm [20]. A comparison of all three models on data from *C. reinhardtii* favours curvature control [24]. Despite years of theoretical effort, it is not clear if any of these feedback mechanisms are involved in producing the flagellum beat.

In Bayly & Dutcher [25], an alternative mechanism was proposed and analysed for producing the flagellum beat that does not require dynein regulation or spatiotemporal organization of dynein activity. The mechanism suggested in [25] is related to a dynamic instability known as flutter, which results when an elastic structure in fluid is subject to axial loading. Dynein generates a tension that buckles the filament. Unlike static buckling, the direction of the motor forces remains tangent to the filament as it deforms, which results in an oscillatory motion.

The instability analysed in [25] is similar to the instability of filaments under external load in the tangent direction known as a ‘follower force’ (because it follows the direction of the filament) [26]. There have been several recent analyses of filaments subjected to a follower force at a low Reynolds number inspired by the motion of biological filaments driven by molecular motors [27–33]. These models have been used to understand observations in *in vitro* motility assays [27], cytoplasmic streaming [31], beating flagella and cilia [29], coordination between pairs [32] and arrays of cilia [33]. These works have thoroughly analysed the Hopf bifurcation from rest to a beating pattern including how different boundary conditions and restrictions (i.e. planar versus three-dimensional) result in different dynamics in viscous fluids [28–30].

In this article, we analyse the emergence of oscillations of a planar elastic filament pinned at the base subject to a follower force at the tip in a viscoelastic fluid. We examine how the elasticity of the surrounding fluid affects the strength of the applied force needed to produce the oscillation and the emergent frequency at the bifurcation. Our results show that the critical value of the force at which oscillations occur is greater in a viscoelastic fluid than in a Newtonian fluid, and the frequency of the beat is always increased by the elasticity of the fluid. We compare the model predictions for how the frequency changes with relaxation time and total viscosity with the experimental measurements from Qin *et al*. [8]. Our analysis captures the observed frequency increases with relaxation time and the non-monotonic frequency response to changes in viscosity, and thus offers a possible mechanistic explanation for how the beat frequency is affected by fluid elasticity.

## 2. Model and equations

**Figure 1.**
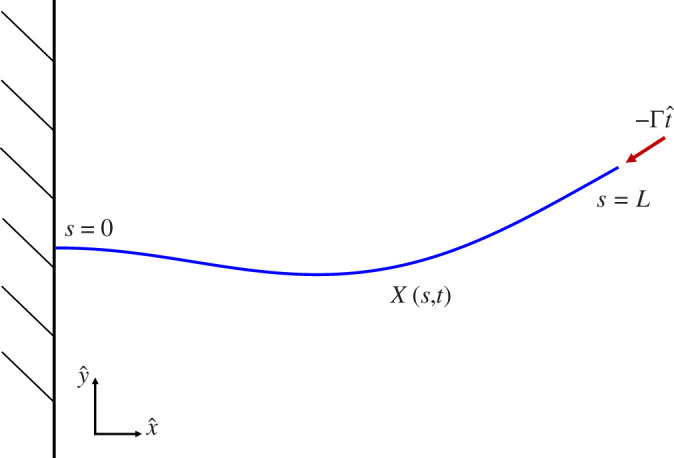
Schematic of a horizontal flexible filament clamped at one end with a follower force of strength Γ applied at its tip. The filament position is defined as X(s,t) , where 0≤s≤L is the arc length coordinate. The local tangent vector is $X_{s}=\hat{t}(s,t)$ .

We consider the motion of a slender, inextensible, planar, elastic filament, clamped at one end and subject to a compressive follower force of strength Γ at the tip of the filament in a viscoelastic fluid at zero Reynolds number. The mathematical model is analogous to that presented in [28] with the addition of fluid viscoelasticity. Let 0≤s≤L be the arclength, where s=0 corresponds to the clamped base. The position of the filament is X(s,t)=(x(s,t),y(s,t)) ; see figure 1.

The instantaneous force balance for the filament is


(2.1) $$-k_{b}X_{ssss}-(TX_{s}{)}_{s}+F^{\text{fluid}}=0,$$

where the first term is the force per unit length from bending, the second term represents the tension that enforces the inextensibility constraint $|X_{s}|=1$ , and $F^{\text{fluid}}$ is the drag force from the surrounding fluid. We assume that the fluid drag can be expressed as the drag in a viscous fluid plus a drag accounting for the viscoelastic effects. One can think of the fluid as composed of a Newtonian solvent with the addition of polymers which are responsible for viscoelastic stresses. Thus, the drag force is


(2.2) $$F^{\text{fluid}}=F^{\text{sol}}+F^{\text{pol}},$$

where $F^{\text{sol}}$ and $F^{\text{pol}}$ are the drag forces owing to the solvent and polymers, respectively.

At the clamped end, we have the boundary conditions


(2.3) $$X(0,t)=0,\;\;\;\;\;\text{ and }\;\;\;\;\;X_{s}(0,t)={\hat{e}}_{x},$$

while at the free end


(2.4) $$\begin{array}{rl} & X_{ss}(L,t)=0, \end{array}$$

(2.5) $$\begin{array}{rl} & -k_{b}X_{sss}(L,t)-T(L,t)X_{s}(L,t)=-\Gamma X_{s}(L,t), \end{array}$$

which captures the fact that the filament is torque-free and that the force at the tail and the external force must balance. The compressive follower force, $\Gamma X_{s}$ , is applied tangentially to the tip of the filament. This non-conservative force drives the motion of the filament.

The viscous drag force owing to the solvent $F^{\text{sol}}$ acting on the filament from the surrounding flow is given by resistive force theory [34] which provides a local relation between the local filament velocity, $X_{t}$ , and the hydrodynamic force per unit length. The viscous drag force per unit length is defined as


(2.6) $$F^{\text{sol}}=-(\xi_{||}^{s}\hat{t}\hat{t}+\xi_{⊥}^{s}\hat{n}\hat{n})\cdot X_{t},$$

where $\hat{t}$ and $\hat{n}$ are the local tangent and normal unit vectors, respectively. The drag coefficients in the perpendicular and parallel directions, $\xi_{⊥}^{s}$ and $\xi_{||}^{s}$ , are proportional to the solvent viscosity $\mu_{s}$ . For example, for a cylinder of radius b and length L , $\xi_{⊥}^{s}=\mu_{s}\alpha$ , where α=4π/[ln⁡(L/b)+1/2] and $\xi_{⊥}^{s}/\xi_{||}^{s}\to 2$ as L/b→∞ [34].

We are interested in the effects of viscoelasticity at and near the bifurcation at which oscillations first emerge, and are thus small in amplitude. Given this, we use a linear viscoelastic model to describe the polymeric force $F^{\text{pol}}$ :


(2.7) $$\tau F_{t}^{\text{pol}}+F^{\text{pol}}=-(\xi_{||}^{p}\hat{t}\hat{t}+\xi_{⊥}^{p}\hat{n}\hat{n})\cdot X_{t},$$

where $\xi_{⊥}^{p}=\mu_{p}\alpha$ , $\mu_{p}$ is the polymeric viscosity, and τ is the fluid relaxation time. In the limit of zero fluid relaxation time ( τ=0 ) or zero polymer viscosity $\left(\mu_{p}=0\right)$ , the polymeric force $F^{\text{pol}}=0$ and we recover the viscous Newtonian fluid. The assumption of a linear viscoelastic model for the drag force on a deforming filament at a small amplitude was used by previous authors to analyse the effect of viscoelasticity on shape changes of flagellum shapes and bending filaments [12,13]. Furthermore, this assumption was numerically validated in [13] by comparing this linear model with numerical simulations that involve the nonlinear viscoelastic stress.

Equations (2.1), (2.6) and (2.7) are non-dimensionalized by rescaling lengths by L , time by the viscous timescale $L^{4}(\xi_{⊥}^{s}+\xi_{⊥}^{p})/k_{b}$ , tension (the Lagrangian multiplier) by $k_{b}/L^{2}$ and polymeric force by $k_{b}/L^{3}$ , yielding


(2.8) $$\begin{array}{rl} & -X_{ssss}-(\hat{T}X_{s}{)}_{s}-\beta (R\hat{\text{t}}\hat{\text{t}}+\hat{\text{n}}\hat{\text{n}})X_{t}+F^{\text{pol}}=0, \end{array}$$

(2.9) $$\begin{array}{rl} & \lambda F_{t}^{\text{pol}}+F^{\text{pol}}=-(1-\beta )(R\hat{\text{t}}\hat{\text{t}}+\hat{\text{n}}\hat{\text{n}})X_{t}. \end{array}$$

Here, $\hat{T}=TL^{2}/k_{b}$ is the dimensionless tension, $\beta =\mu_{s}/(\mu_{s}+\mu_{p})$ is the viscosity ratio of the viscoelastic fluid, $R=\xi_{||}^{s}/\xi_{⊥}^{s}=\xi_{||}^{p}/\xi_{⊥}^{p}$ is the ratio of tangential and normal drag coefficients and $\lambda =\tau k_{b}/L^{4}(\xi_{⊥}^{s}+\xi_{⊥}^{p})$ is the dimensionless relaxation time. Note that the ratio of tangential and normal drag coefficients depends only on the aspect ratio of the filament, not on the viscosity, and thus has the same value for the solvent and polymeric fluid.

Similarly, rescaling equations (2.4) and (2.5) yields the following dimensionless free boundary conditions:


(2.10) $$\begin{array}{rl} & X_{ss}(1,t)=0, \end{array}$$

(2.11) $$-X_{sss}(1,t)-\hat{T}(1,t)X_{s}(1,t)=-\sigma X_{s}(1,t),$$

where the dimensionless ratio between the strength of the force at the tip and the elastic force is defined as


(2.12) $$\sigma =\Gamma L^{2}/k_{b}.$$

Since the force is compressive (Γ>0) , σ is always positive.

## 3. Bifurcation analysis in a viscoelastic fluid

In a viscous fluid, the strength of the follower force, σ , is the only non-dimensional parameter. As analysed in [28], there is a critical strength of the follower force, $\sigma_{0}$ , below which the straight filament at rest is stable. At $\sigma =\sigma_{0}$ , there is a supercritical Hopf bifurcation, so that for $\sigma >\sigma_{0}$ , the filament exhibits sustained oscillations. In a viscoelastic fluid, there are three dimensionless parameters to consider: the follower force strength, σ , the relaxation time, λ , and the viscosity ratio, β . In this section, we analyse how the critical follower strength and the frequency of the emergent oscillation in a viscoelastic fluid depend on λ and β .

We consider small amplitude deviations from the rest state of a straight filament. In the small deformation regime, the tension to the leading order is constant and therefore equal to the external force applied to the tip [19,28], so that $\hat{T}(s,t)=\sigma$ . The leading order equations for the vertical displacement, y(s,t) , and vertical component of the polymer force, $f^{\text{pol}}(s,t)$ , are


(3.1) $$-\beta y_{t}-y_{ssss}-\sigma y_{ss}+f^{\text{pol}}=0,$$

(3.2) $$\lambda f_{t}^{\text{pol}}+f^{\text{pol}}=-(1-\beta )y_{t}.$$

Deformations in the horizontal direction and changes to the tension occur at higher order in deformation. The resulting boundary conditions are


(3.3) $$y(0,t)=y_{s}(0,t)=y_{ss}(1,t)=y_{sss}(1,t)=0.$$

### 3.1. Relationship to stability in a viscous fluid

We assume solutions of the form $y(s,t)=\hat{y}(s)e^{\eta_{\text{ve}}t}$ and $f^{\text{pol}}(s,t)={\hat{f}}^{\text{pol}}(s)e^{\eta_{\text{ve}}t}$ in Equation (3.1), (3.2) and (3.3). The real part of $\eta_{\text{ve}}$ quantifies the growth (or decay if negative) rate of perturbations in a viscoelastic fluid, and its imaginary part gives the frequency of oscillations.

Eliminating ${\hat{f}}^{\text{pol}}$ we obtain


(3.4) $$\begin{array}{l} -{\hat{y}}_{ssss}-\sigma {\hat{y}}_{ss}=\left(\beta \eta \text{ve}+\frac{(1-\beta )\eta \text{ve}}{\left(\lambda \eta \text{ve}+1\right)}\right)\hat{y} \end{array}.$$

We let $L=-\partial_{ssss}-\sigma \partial_{ss}$ denote the operator acting on the space of functions that satisfy boundary conditions (3.3). We express (3.4) as the eigenvalue problem


(3.5) $$L\hat{y}=\eta_{\text{v}}\hat{y},$$

where


(3.6) $$\eta_{\text{v}}=\frac{(1-\beta )\eta_{\text{ve}}}{\lambda \eta_{\text{ve}}+1}+\beta \eta_{\text{ve}}$$

denotes the eigenvalues of L , the real part of which quantifies the growth rate of perturbations in a viscous fluid. Consistent with this idea, note that when either β=1 or λ=0 , $\eta_{\text{ve}}=\eta_{\text{v}}$ because the viscoelastic fluid reduces to a viscous fluid.

#### 3.1.1. Instability in a viscous fluid is necessary for instability in a viscoelastic fluid

We first show that for a given strength of the follower force, the system is unstable in a viscoelastic fluid only if the system is unstable in a viscous fluid. Equation (3.6) relating the eigenvalues of the follower problem in the viscoelastic fluid to those in a viscous fluid can be expressed as


(3.7) $$\eta_{\text{v}}=\frac{(1-\beta )\lambda |\eta_{\text{ve}}{|}^{2}}{{\left|1+\lambda \eta_{\text{ve}}\right|}^{2}}+\left(\frac{\left(1-\beta \right)}{{\left|1+\lambda \eta_{\text{ve}}\right|}^{2}}+\beta \right)\eta_{\text{ve}}.$$

The real parts of $\eta_{\text{v}}$ and $\eta_{\text{ve}}$ are related by


(3.8) $$Re(\eta_{\text{v}})=\alpha_{0}\left(\eta_{\text{ve}}\right)+\alpha_{1}\left(\eta_{\text{ve}}\right)Re(\eta_{\text{ve}}),$$

where $\alpha_{0}$ and $\alpha_{1}$ are real valued, non-negative functions of $\eta_{\text{ve}}$ . Therefore, if $Re(\eta_{\text{ve}})>0$ , then $Re(\eta_{\text{v}})>0$ . This establishes that instability in a viscous fluid is a necessary condition for instability in a viscoelastic fluid.

#### 3.1.2. More force is required for instability in viscoelastic fluid

Let $\hat{\sigma }$ denote the value of the follower force at which the rest state becomes unstable in the viscoelastic fluid. Because $Re(\eta_{\text{ve}})=0$ when $\sigma =\hat{\sigma }$ , $Re(\eta_{\text{v}})=c_{0}\left(\eta_{\text{ve}}\right)>0$ from (equation 3.8). Because $Re(\eta_{\text{v}})<0$ for all $\sigma <\sigma_{0}$ , it follows that $\hat{\sigma }>\sigma_{0}$ . Therefore, it follows that the follower force required for instability in the viscoelastic case is always greater than the force required in the viscous case.

#### 3.1.3. Higher frequency in viscoelastic fluid

We show that for the same follower force, the frequency of the oscillation in the viscoelastic fluid is always greater than the frequency in the viscous fluid. From equation (3.7), the imaginary parts of $\eta_{\text{v}}$ and $\eta_{\text{ve}}$ are related by


(3.9) $$Im(\eta_{\text{v}})=\left(\frac{\left(1-\beta \right)}{{\left|1+\lambda \eta_{\text{ve}}\right|}^{2}}+\beta \right)Im(\eta_{\text{ve}}).$$

Assume that $Re(\eta_{\text{ve}})>0$ , so that the rest state is unstable. If it follows that ${\left|1+\lambda \eta_{\text{ve}}\right|}^{2}>1$ , and thus from equation (3.9)


(3.10) $$Im(\eta_{\text{v}})<Im(\eta_{\text{ve}}).$$

Because the emergent frequency of the oscillation near the bifurcation is approximately the imaginary part of the eigenvalue, we conclude that viscoelasticity increases the frequency of the oscillation.

### 3.2. Numerical calculation of eigenvalues

**Figure 2.**
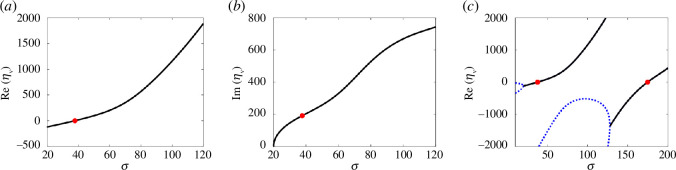
Real part (*a*) and imaginary part (*b*) of the eigenvalue with the largest real part for a viscous fluid as a function of the follower force, σ . (*c*) The real part of the four eigenvalues with the largest real parts. Blue dashed lines denote real eigenvalues, solid black lines denote pairs of complex eigenvalues and red dots mark where the real part changes sign.

We obtain the viscous eigenvalues using a second-order, centred finite-difference discretization of the operator L appropriately modified near the ends to account for the boundary conditions. The real and imaginary parts of the eigenvalue with the largest real part are plotted in figure 2*a*,*b*. Consistent with De Canio *et al*. [28], we find the critical strength of the follower force at which oscillations emerge in the viscous fluid is $\sigma_{0}\approx 37.7$ . At this critical value, the eigenvalues corresponding to the bifurcation are $\eta_{\text{v}}\approx \pm 191i$ . We define $\omega_{0}\approx 191$ as the angular frequency of the emergent oscillation in a viscous fluid.

Given the viscous eigenvalues as a function of σ , for a given relaxation time, λ , and viscosity ratio, β , we identify the corresponding viscoelastic eigenvalues by solving equation (3.6) for $\eta_{\text{ve}}$ . To identify instability in the viscoelastic case, we need to compute only the viscoelastic eigenvalues when the corresponding viscous eigenvalues have a positive real part. We find that for $\sigma_{0}<\sigma <174.6$ , there is only a single pair of eigenvalues with a positive real part for the viscous fluid as shown in figure 2*c*. In the remainder of this work, we restrict σ<174.6 which simplifies the stability analysis in the viscoelastic case because we need to consider only the viscoelastic eigenvalues corresponding to a single viscous eigenvalue.

### 3.3. Asymptotic analysis of instability in a viscoelastic fluid

#### 3.3.1. Limit of a large relaxation time

As λ→∞ equation (3.6) at the leading order is


(3.11) $$\eta_{\text{v}}=\beta \eta_{\text{ve}}+O\left(\lambda^{-1}\right)$$

Thus, in this limit of large relaxation time, the viscoelastic eigenvalue is proportional to the viscous eigenvalue, and two conclusions follow immediately from this relation. First, the critical follower force strength at which oscillations emerge in the viscoelastic fluid (i.e. the Hopf bifurcation point) is identical to the critical follower force for a viscous fluid. Second, the emergent frequencies in the two fluids are related by


(3.12) $$\omega_{\text{v}e}=\beta^{-1}\omega_{\text{v}}.$$

Because 0<β≤1 , the frequency in the viscoelastic case is always greater than the frequency in the viscous case. In summary, in the limit λ→∞ , the bifurcation occurs at the same follower force strength and the emergent frequency is greater in the viscoelastic case by a factor inversely proportional to the viscosity ratio. We note that many biologically relevant media such as respiratory and cervical mucus have relatively large relaxation times [35].

#### 3.3.2. Limit of vanishing polymer viscosity: β→1

There are two limits in which the viscoelastic fluid reduces to a viscous fluid: vanishing relaxation time ( λ→0 ) and vanishing polymer viscosity ( β→1 ). Both limits are useful in considering perturbation from a viscous fluid. We consider the limit β→1 , which physically corresponds to the limit of small polymer viscosity and is typical of dilute polymeric solutions. In this limit, we are able to examine how the critical follower force strength and emergent frequency depend on both the relaxation time and follower force strength for all relaxation times. As we explain later, this analysis also captures the limit λ→0 .

At the bifurcation point, the viscoelastic eigenvalue is pure imaginary, i.e. $\eta_{\text{ve}}=i\omega$ , where ω is the angular frequency at the bifurcation point. The relationship between the viscous and viscoelastic eigenvalues, equation (3.6), at the bifurcation point is


(3.13) $$\eta_{\text{v}}\left(\sigma \right)=\frac{(1-\beta )i\omega }{\lambda i\omega +1}+\beta i\omega ,$$

where the notation $\eta_{\text{v}}\left(\sigma \right)$ is used to emphasize that the viscous eigenvalue depends on the unknown follower force strength, σ . This is a single complex-valued equation involving the two real-valued unknowns σ and ω .

For β close to 1, σ will be close to $\sigma_{0}$ , the critical follower force strength in a viscous fluid. We linearize $\eta_{\text{v}}$ about this point so that


(3.14) $$\eta_{\text{v}}\left(\sigma \right)=i\omega_{0}+\hat{\sigma }(a+bi)+O\left({\hat{\sigma }}^{2}\right),$$

where $\hat{\sigma }=\sigma -\sigma_{0}$ and ${(d\eta_{\text{v}}/d\sigma )|}_{\sigma_{0}}=a+bi$ . Eliminating $\eta_{\text{v}}$ from equation (3.13) using equation (3.14) and then equating the real and imaginary parts results in


(3.15) $$\begin{array}{rl} a\hat{\sigma } & =\frac{\epsilon \omega^{2}\lambda }{1+\omega^{2}\lambda^{2}}+O({\hat{\sigma }}^{2}), \end{array}$$

(3.16) $$\begin{array}{rl} \omega_{0}+\hat{\sigma }b & =\omega (1-\epsilon \frac{\omega^{2}\lambda^{2}}{1+\omega^{2}\lambda^{2}})+O({\hat{\sigma }}^{2}), \end{array}$$

where ϵ=1−β . We seek a solution to the limit ϵ→0 by using the expansions $\hat{\sigma }=\epsilon \sigma_{1}+\epsilon^{2}\sigma_{2}+\cdots$ and $\omega =\omega_{0}+\epsilon \omega_{1}+\cdots$ and matching the O(ϵ) terms. The resulting expansions for critical follower force and corresponding frequency are


(3.17) $$\begin{array}{rl} \sigma & =\sigma_{0}+(1-\beta )\frac{\lambda \omega_{0}^{2}}{a\left(1+\lambda^{2}\omega_{0}^{2}\right)}+O((1-\beta {)}^{2}), \end{array}$$

(3.18) $$\begin{array}{rl} \omega & =\omega_{0}+(1-\beta )\omega_{0}\left(\frac{\frac{b}{a}\lambda \omega_{0}+\lambda^{2}\omega_{0}^{2}}{1+\lambda^{2}\omega_{0}^{2}}\right)+O((1-\beta {)}^{2}). \end{array}$$

In these expressions, the relaxation time appears paired with the frequency of the product $\lambda \omega_{0}$ . This quantity is similar to the Deborah number, but the frequency is fixed at the emergent frequency in the viscous limit. In this section, we consider how the bifurcation location and emergent frequency depend on the scaled relaxation time $\lambda \omega_{0}$ .

Before continuing, we remark that one can recover the λ→0 expansions from these expressions. Equations 3.15 and 3.16 are valid near the viscous bifurcation point, and thus when λ→0 for all β . Equation (3.17) follows directly from equation (3.15). The expressions for the frequency in the limit λ→0 that one obtains from expanding equation (3.16) or equation (3.18) are equivalent at O(λ) .

**Figure 3.**
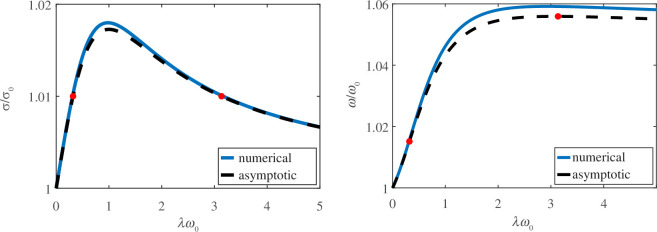
Plots of the asymptotic and numerical solutions as β→1 for the (*a*) critical follower force and (*b*) emergent frequency at the bifurcation as functions of the relaxation time for β=0.95 . The two red dots mark the bifurcation points and emergent frequency, respectively, for $\sigma =1.01\sigma_{0}$ .

In figure 3, we plot the asymptotic and numerical solutions for critical follower force and emergent frequency in a viscoelastic fluid relative to their respective values in a viscous fluid for β=0.95 as a function of relaxation time. Plots for other values of β are shown in the electronic supplementary material [36]. As the relaxation time increases, the critical force increases and then decreases. The peak critical force occurs at $\lambda \omega_{0}=1$ , and as expected from the large relaxation time analysis $\sigma \to \sigma_{0}$ as λ→∞ .

The emergent frequency also increases and then decreases as it approaches a frequency greater than the corresponding viscous frequency, again, as expected. However, note that $\omega /\omega_{0}\to \beta^{-1}$ according to equation (3.11) and $\omega /\omega_{0}\to 1+(1-\beta )$ according to equation (3.18). Because $\beta^{-1}=1+(1-\beta )+O(1-\beta )$ , these expressions are not inconsistent. The peak in the frequency occurs at a larger relaxation time than the peak in the critical force. Specifically, the peak occurs at $\lambda \omega_{0}=a/b+\sqrt{a^{2}/b^{2}+1}\approx 3.07$ .

For small polymer viscosity, there remains a single critical value of the follower force above which oscillations emerge. However, for the range of follower forces below the peak in figure 3*a* (which is approximately $1<\sigma /\sigma_{0}<1+(1-\beta )/(2a\sigma_{0})$ ), there are two Hopf bifurcation points as the relaxation time changes. For a follower force in this range, the filament oscillates at low and high relaxation times while the rest state is stable for an intermediate range of relaxation times. The frequency at the higher relaxation time is generally greater than that at the lower relaxation time. For example, for β=0.95 and $\sigma =1.01\sigma_{0}$ , the two bifurcation points and critical frequencies are marked with red dots in figure 3. In later sections, we examine how the frequency changes with relaxation time for fixed follower force, and we will see that generally the frequency increases with increasing relaxation time.

### 3.4. Bifurcation location and emergent frequency for general β

**Figure 4.**
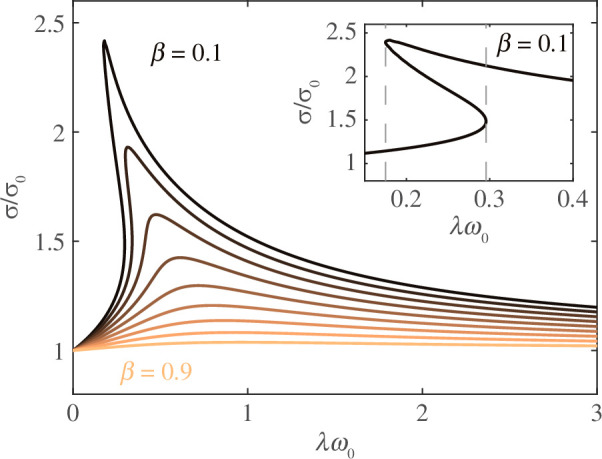
Location of the bifurcation point in the σ - $\lambda \omega_{0}$ plane for β=0.1,0.2,…,0.9 . Inset: Location of the bifurcation for β=0.1 zoomed in to show that between the two dashed vertical lines there are three bifurcation points as the follower force changes.

We examine the bifurcation for general values of the viscosity ratio β by solving equation (3.6) for $\eta_{\text{ve}}$ using the numerically computed viscous eigenvalues. Note that there are two values of $\eta_{\text{ve}}$ for each viscous eigenvalue. We found that for all parameter regimes we explored, one of the two values of $\eta_{\text{ve}}$ always had a negative real part. As discussed in the electronic supplementary material [36], this additional eigenvalue scales with 1/λ and is likely related to the fluid relaxation timescale. In figure 4, we show the location of the bifurcation in the σ - $\lambda \omega_{0}$ plane for β=0.1,0.2,…,0.9 . For many values of β , the shape of the curve denoting the location of the bifurcation is qualitatively similar to that of the asymptotic result. In the limit β→1 , the peak in the critical follower force occurs at $\lambda \omega_{0}=1$ , but as β decreases, the relaxation time corresponding to this peak decreases.

For example for β=0.5 , the peak occurs near $\lambda \omega_{0}\approx 0.72$ . For small values of β , the critical force strength is no longer a single-valued function of the relaxation time. The inset in figure 4 shows the bifurcation location for β=0.1 for a smaller range of λ to highlight this feature. At β=0.1 for $0.1749<\lambda \omega_{0}<0.2955$ (end points marked with grey lines in the figure), there are three σ values at which bifurcations occur.

**Figure 5.**
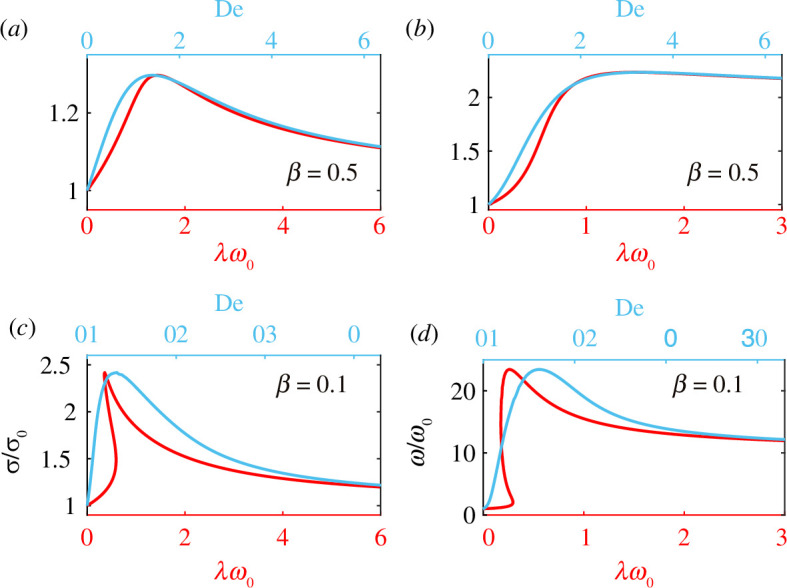
Bifurcation location (*a,c*) and frequency (*b*,*d*) at the bifurcation for β=0.5 (*a*,*b*) and (*c*,*d*) β=0.1 . The two curves in each panel represent two different scalings for the relaxation time, λ . The red curves (bottom axes) show how the data depend on $\lambda \omega_{0}$ , where $\omega_{0}$ is the angular frequency at the bifurcation point in a viscous fluid. The blue curves (top axes) show the data depending on the Deborah number De=λω , where ω is the emergent frequency in a viscoelastic fluid. Because the emergent frequency depends on the relaxation time, De represents a non-uniform scaling of the relaxation time.

The quantity $\lambda \omega_{0}$ that appears in the asymptotic expressions (3.17) and (3.18) represents the Deborah number in the limit of vanishing polymer viscosity ( β→1 ). More generally, we take as the Deborah number the product of the relaxation time and the emergent frequency, i.e. De=λω . In figure 5, we examine how the critical force and the emergent frequency depend on $\lambda \omega_{0}$ and De=λω for β=0.5 and β=0.1 . For β=0.5 , the shapes of the critical force and emergent frequency are qualitatively similar when viewed as functions of either $\lambda \omega_{0}$ or De . However, for β=0.1 , there is a substantial difference in these curves. The critical force and emergent frequency are single-valued functions of De . Also, the shapes of these curves as functions of De are qualitatively similar to the shapes predicted by the asymptotic analysis as β→1 and corresponding curves for β=0.5 . Both the critical force and the emergent frequency are multi-valued in the same range of relaxation times ( $0.1749<\lambda \omega_{0}<0.2955$ for β=0.1 ). For each value of λ in this range, there are three different bifurcation points each with its own frequency and hence its own distinct Deborah number. This explains why the critical force and emergent frequency are single-valued functions of De .

In later results, we use both the scaled relaxation time $\lambda \omega_{0}$ and the Deborah number λω . The former is particularly useful when examining how quantities change with relaxation time. Because the emergent frequency depends on the relaxation time, the Deborah number is not simply proportional to the relaxation time.

## 4. Frequency analysis for a fixed follower force in a viscoelastic fluid

### 4.1. Frequency changes for varying relaxation times

In the previous section, we examined how the emergent frequency at the bifurcation depends on the fluid parameters. However, the bifurcation location depends on the fluid parameters. Hence, as the fluid parameters vary, the follower force varies as well. Here, we explore how the emergent frequency depends on fluid elasticity and viscosity at a *fixed* follower force. Close to the bifurcation, the angular frequency is approximately the imaginary part of the eigenvalue with a positive real part. In order for the results from linear stability analysis to be relevant, we consider solutions that are close to the bifurcation. For a fixed β , the critical force where oscillations emerge is non-monotonic in λ, and there is a maximum critical force as a function of λ , e.g. see figure 4. We choose the force to be approximately 1% higher than the maximum for a particular value of β and examine how the frequency changes as a function of λ . This choice of force is large enough to avoid bifurcations in λ where the oscillations cease and small enough to avoid large-amplitude motion where the linear analysis is less accurate. For example, for β≥0.5 the local maximum in force $\sigma ≲1.3\sigma_{0}$ which is still relatively close to the bifurcation for all relaxation times.

**Figure 6.**
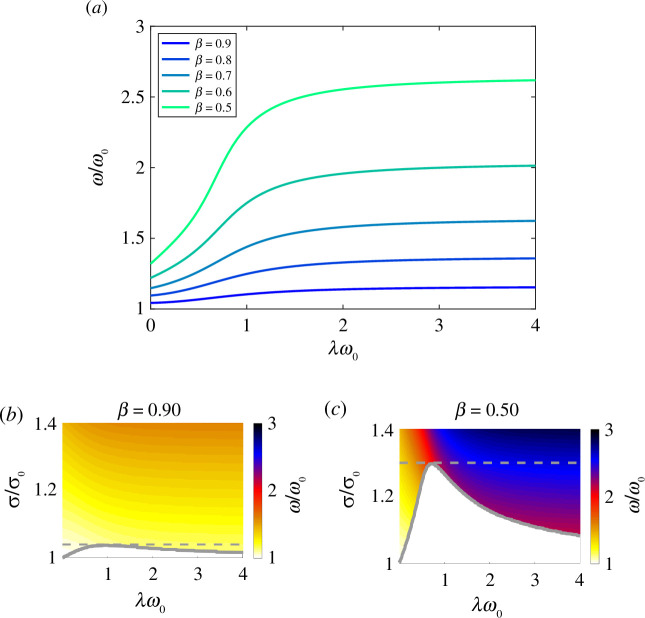
Emergent frequency (*a*) as a function of scaled relaxation time for β≥0.5 at a fixed follower force that is 1% higher than the maximum force at the bifurcation for the corresponding β . Colour fields of frequency for β=0.9 (*b*) and β=0.5 (*c*). Solid lines show the location of bifurcation, and the dashed line shows the fixed force value for the corresponding frequency values shown above.

In figure 6*a*, we show the emergent frequency scaled by $\omega_{0}$ at a fixed follower force as a function of the scaled relaxation time $\lambda \omega_{0}$ for a range of β≥0.5. The emergent frequency is monotonically increasing for a fixed follower force for each β , and the frequency increase levels off for large $\lambda \omega_{0}$ . The emergent frequency also increases with decreasing β. For β=0.9 , the frequency at $\lambda \omega_{0}=4$ is about 10% higher than the viscous frequency at the same force, whereas for β=0.5 , the frequency at $\lambda \omega_{0}=4$ is nearly double the viscous frequency at the same force.

In figure 6*b,c*, we show colour fields of the emergent frequency as a function of both $\lambda \omega_{0}$ and $\sigma /\sigma_{0}$ for β=0.9,0.5. The fixed follower force strength corresponding to figure 6 is highlighted with grey-dashed lines. For follower forces fixed at higher values (above the grey line), the qualitative behaviour of the frequency is the same, namely, the frequency increases rapidly for $\lambda \omega_{0}≲1$ and levels off for higher relaxation times. Quantitatively, higher forces lead to higher frequencies overall.

### 4.2. Comparing analysis and numerical simulations

To explore how well the linear stability analysis predicts the emergent frequencies away from the bifurcation, we solve equations (2.8) and (2.9) numerically with boundary conditions given by equations (2.10) and (2.11), which accounts for both normal and tangential deformations. Details of the numerical method are described in the electronic supplementary material [36]. With these simulations, we are also able to examine how the amplitude and shape change with varying fluid rheology.

**Figure 7.**
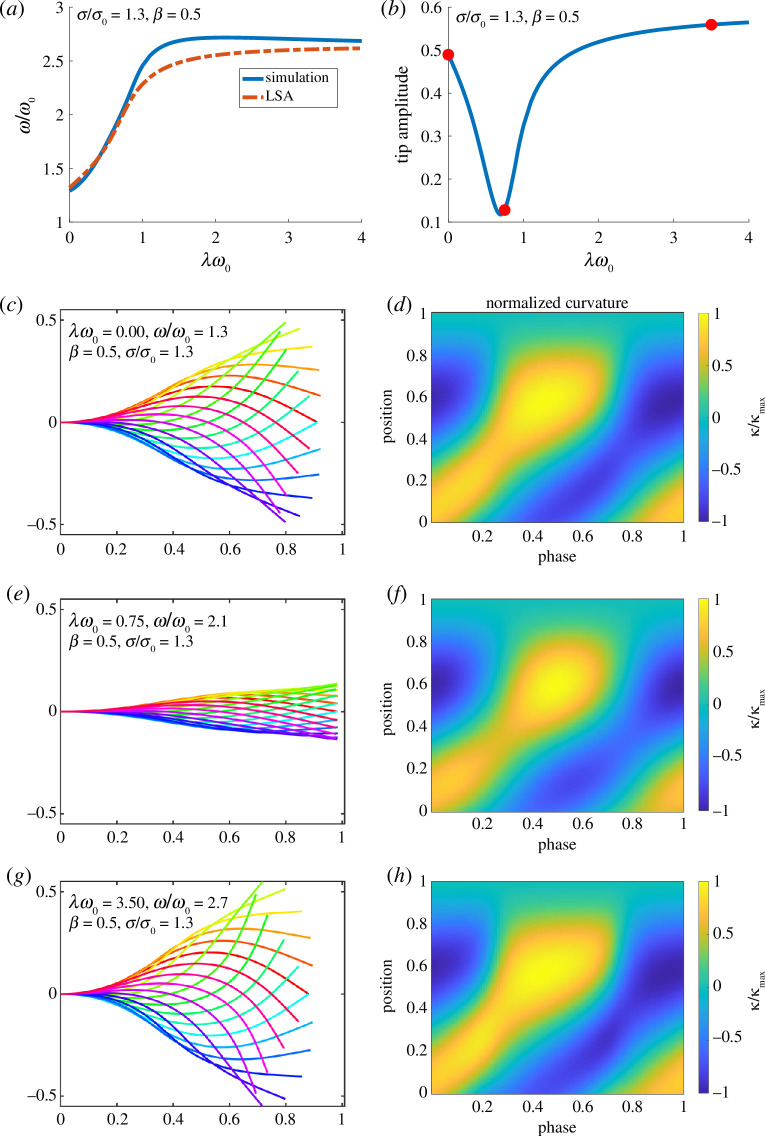
Emergent frequency (*a*) and tip amplitude (*b*) for $\beta =0.5,\sigma /\sigma_{0}=1.3.$ Frequency figure shows a comparison with simulation and linear stability analysis. Emergent shapes with 20 snapshots per period (*c*,*e*,*g*) and curvature normalized by its maximum ( $\kappa /\kappa_{\text{max}}$ ) (*d*,*f*,*h*) over a period for simulations with $\lambda \omega_{0}=0,0.75,3.5$ , these values are highlighted with red dots above. The maximum values of curvature for these three values of $\lambda \omega_{0}$ are 3.01 , 1.15 and 3.56 , respectively. The amplitude of the curvature follows the same trend as the tip amplitude.

In figure 7*a*, we compare the results for emergent frequency in the linear stability analysis and the simulations for $\beta =0.5,\sigma /\sigma_{0}=1.3.$ The corresponding amplitude of the tip of the filament is shown in figure 7*b*. Despite the fact that the amplitude is not particularly small, the simulations qualitatively match the frequency predicted from the linear stability analysis, with the simulations exhibiting slightly higher frequencies for moderate $\lambda \omega_{0}.$ The amplitude of the oscillation is not available from the linear analysis, but the amplitude is related to the distance from the bifurcation. Because the oscillation emerges at a Hopf bifurcation, the amplitude should grow like $(\sigma -\sigma_{c}{)}^{1/2}$ , where $\sigma_{c}$ represents the follower force strength at the bifurcation. The fact that the amplitude initially decreases and then increases to a constant value as it increases is consistent with how the distance to the bifurcation changes.

In figure 7*c–h*, shapes (left) and normalized curvatures (right) for the simulations at $\lambda \omega_{0}=0,0.75,3.5$ (corresponding to the red dots on the amplitude figure above) are shown. The plots in figure 7*c*,*e,g* show the filaments at the same phase in the period (phase is labelled by colour), and the actual frequency of motion is listed in the figure. Other than changes in amplitude and frequency, the overall shapes are similar. This can be seen more clearly in the kymographs of curvature normalized by its maximum value in figure 7*d*,*f,g*. The peak values of curvature are 3.01 , 1.15 and 3.56 for $\lambda \omega_{0}=0$ , 0.75 and 3.50 , respectively. The amplitude of the curvature follows the same trend as the tip amplitude, which as noted above, is related to the distance from the bifurcation. The curvatures exhibit subtle differences but are similar.

Although one may expect more significant shape changes as a function of rheology, the shape of the oscillating filament near the bifurcation is determined by the eigenfunctions of the operator L in equation (3.5). These eigenfunctions do not depend on the fluid elasticity ( λ nor β ). It is only the eigenvalues of the system that change with fluid elasticity. We considered only parameters near the bifurcation point to remain in the low-amplitude regime so that linear viscoelasticity was a reasonable approximation. Other analyses of filaments subject to follower forces in viscous fluids demonstrated significant shape variation and different kinds of motion at large amplitudes that depend on the boundary conditions and distribution of follower forces [29,30]. There may be shape changes owing to fluid elasticity at higher amplitudes, but at large amplitude, one must consider viscoelastic nonlinearity [13,37].

### 4.3. Comparing frequency changes with experiments

In Qin *et al*. [8], the flagellar beat pattern, beat frequency and swimming speed of the bi-flagellated alga *C. reinhardtii* were measured in response to systematic variation of the fluid viscosity and relaxation time (or fluid elasticity). Surprisingly, it was observed that the beat frequency increased with increasing relaxation time. In a Newtonian fluid, the beat frequency decreased monotonically with the fluid viscosity, but in a viscoelastic fluid, the frequency changed non-monotonically with the fluid viscosity, initially decreasing, then increasing and appearing to plateau. The physical origins of the frequency response to fluid elasticity are not currently understood. Here, we examine the predictions of the follower model to compare with these experimental observations.

It is reasonable to ask whether one expects the predictions from the analysis of the follower model to be relevant to flagella which are driven by dynamic motor forces along the filament. If the motor activity is constant or does not change with the rheology of the fluid, then the predictions of our analysis that follow from the relationship between the eigenvalues in equation (3.6) will hold. Namely, the analysis in §3 predicts a higher beat frequency in a viscoelastic fluid, which approaches a constant as the relaxation time increases. Several studies have examined different mechanisms of motor feedback and control in the linearized equations, and these models are capable of matching experimental data [19,20,24]. While there are different proposed control mechanisms, all of them are assumed to depend on the frequency of the emergent beat. In the electronic supplementary material [36], we show that even when the motor activity changes with emergent frequency, in the limit of vanishing polymer viscosity ( β→1 ), the expression for the frequency at the bifurcation is of the same form as equation (3.18). Thus, it is expected that the frequency generally increases with relaxation time and approaches a constant value in the limit of large relaxation time. As we show below, these two features of the frequency response to fluid elasticity are consistent with the data from Qin *et al*. [8], and thus our analysis may provide insight into the mechanisms underlying the observations.

The viscoelastic fluids in [8] were prepared by adding small amounts of the high molecular weight, flexible polymer polyacrylamide to water. The addition of the polymer not only increases the fluid relaxation time but also changes the total viscosity. In terms of the parameters used in this work, both β and λ change simultaneously.

In order to compare the predictions of the follower model with these experiments, we fit the rheological data from Qin *et al*. [8] to find functional forms for the polymer viscosity, $\mu_{p}(c)$ , and the (dimensional) relaxation time, τ(c) , as functions of the polymer concentration c in parts per million. Details of our fitting procedure are given in the electronic supplementary material [36]. Using these fits and the non-dimensionalization in §2, we obtain the dimensionless relaxation time and solvent fraction $\lambda (c)=\tau (c)k_{b}/(L^{4}(\mu_{s}+\mu_{p}(c)))$ and $\beta (c)=\mu_{s}/(\mu_{s}+\mu_{p}(c))$ , respectively, as functions of the polymer concentration.

**Figure 8.**
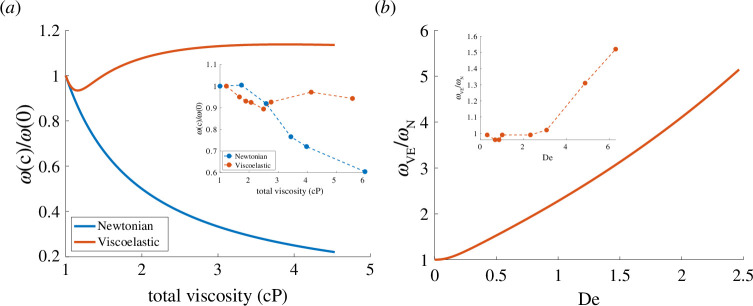
Frequency scaled by its value at c=0 as a function of the total viscosity (*a*). Newtonian viscosity follows 1/viscosity scaling for comparison. Frequency is relative to viscous frequency as a function of the Deborah number (*b*). Simulations run using a fixed value of force $\sigma /\sigma_{0}=1.5$ and $k_{b}/L^{4}=0.25.$ Inset graphs using data from Qin *et al*. [8]

We use these models for λ(c) and β(c) in simulations for a fixed follower force $\sigma /\sigma_{0}=1.5$ and $k_{b}/L^{4}=0.25$ . Other choices for $\sigma /\sigma_{0}$ and $k_{b}/L^{4}$ are considered in the electronic supplementary material [36]. We vary the concentration over the range c=0−80 ppm and compute the dimensional frequency of the oscillation, $\omega_{VE}(c)$ . In figure 8*a*, we plot this frequency normalized by the frequency at c=0 as a function of the total viscosity, $(\mu_{s}+\mu_{p})(c).$ We compare these results with the experimental data from Qin *et al*. [8] plotted in the inset. It is remarkable that frequency changes in a viscoelastic fluid predicted by the follower model agree qualitatively with the experimental data. Specifically, the non-monotonicity of the frequency dependence on viscosity as well as the plateau for high viscosity are all captured by the follower model.

In a Newtonian fluid, the frequency of oscillations in the follower model at a fixed follower force is inversely proportional to the viscosity. This is because in the dimensionless equations, the strength of the follower force is the only parameter, and the timescale in the non-dimensionalization is proportional to the viscosity. In figure 8*a*, we include a plot of the normalized oscillation frequency in a Newtonian fluid: $\omega_{N}(c)/\omega_{N}(0)=(\mu_{s}+\mu_{p}{)}^{-1}$ , and the corresponding experimental data are shown in the inset. Both the follower model and the experimental data show that the frequency decreases with increasing viscosity. However, the follower frequency is inversely proportional to viscosity, but as discussed in [8], the measured frequency in Newtonian fluid scaled like $(\mu_{s}+\mu_{p}{)}^{-1/2}$ for large viscosity, which is consistent with the frequency scaling predicted by a model that includes force-sensitive dynein motor activity [19].

The non-monotonic response of the frequency to viscosity can be explained using the asymptotic analysis in §3.3. For low polymer concentrations, β is close to 1, and the asymptotic expression for the frequency in equation (3.18) holds. Redimensionalizing this expression, the frequency decrease from increasing viscosity occurs at first order in concentration, but the frequency increase from elasticity occurs at second order in concentration. Therefore, for small concentrations, the frequency should drop at the same rate as the frequency in a viscous fluid, as is observed in figure 8*a*. Further, at high polymer concentration, the relaxation time is large, and the frequency is approximately given by equation (3.12). Because β grows at the same rate that the timescale decays, when equation (3.12) is redimensionalized, it predicts that $\omega_{VE}(c)∼\omega_{VE}(0)$ , i.e. the frequency should approach the zero polymer concentration frequency. In figure 8*a*, we see the frequency appears to approach a value of 10% higher than predicted, but recall that equation (3.12) holds at the bifurcation point, and the results in figure 8 are away from the bifurcation. In summary, the analysis predicts that the frequency should initially drop at low polymer concentrations when viscous effects dominate, but it must eventually increase and approach a constant in a regime in which viscous and elastic effects counterbalance each other. These same trends were observed in experiments [8], and thus this analysis provides a possible mechanistic understanding of the observed frequency response to changes in viscosity in viscoelastic fluids.

Viscosity can be varied for both Newtonian and viscoelastic fluids. In order to isolate the effects of elasticity on frequency in [8], the frequency was measured for both Newtonian and viscoelastic fluids at the same total viscosity. The frequency in a viscoelastic fluid relative to the frequency of a viscous fluid of the same viscosity was reported based on the Deborah number De=λω. Similarly, in figure 8*b*, we plot the frequency of the oscillation in a viscoelastic fluid normalized by the corresponding frequency in a Newtonian fluid of the same viscosity as a function of De. We contrast these results with the results in figure 6 for the frequency as a function of the relaxation time for fixed β . In both cases, the frequency increases as the relaxation time increases, but when the viscosity is fixed, as in figure 6, the frequency levels off for a high relaxation time. When the relaxation time and viscosity change together (via the polymer concentration), as in figure 8*b*, the frequency does not approach a constant. These results are, again, qualitatively similar to the experiments from Qin *et al*. [8] (see inset). The agreement between the follower model and the data on *C. reinhardtii* is remarkable given that flagella are powered by dynamic internal molecular motors and the follower model we analysed is driven by a fixed-strength external force. This suggests that the frequency response to fluid elasticity that arises from changes in the fluid drag may apply more generally to systems with different driving forces.

## 5. Discussion

Despite the fact that fluid rheology is known to affect the shape and frequency of beating flagella in many biological systems, there are limited data in which the fluid elasticity is systematically varied, and a mechanistic understanding of how fluid elasticity affects emergent motion does not exist. Theoretical explorations have shown how fluid elasticity can change the shape of the beat with prescribed active forces [12,13], but these models cannot be used to understand the frequency changes observed in experiments [8]. In this article, we extend the model of an elastic filament driven by a follower force at the tip from De Canio *et al*. [28] to examine how fluid elasticity affects the emergence of oscillations and their resulting frequency.

As in a viscous fluid [28], there is a Hopf bifurcation at a critical force at which beating emerges, but unlike the viscous problem in which the critical force is independent of the viscosity, the critical force in a viscoelastic fluid depends on the relaxation time and viscosity ratio. Perhaps it is surprising that fluid elasticity affects the critical force given the similarity of this problem to Euler’s classical buckling problem in which the critical force does not depend on the medium. The classical analysis of Euler’s problem does not consider dynamics, and such an approach is only useful in the case of conservative forces. In general, for the non-conservative follower force problem, the critical load will depend on the medium as well as internal friction [38].

In a viscoelastic fluid, the force required to induce oscillations is always higher than in a viscous fluid. Moreover, there are parameter regions where increasing fluid relaxation time will stabilize an oscillating filament, but upon further increase in the relaxation time, the filament will again oscillate at a higher frequency. Our analysis predicts that fluid elasticity generally increases the frequency of beating over the same filament in a viscous fluid at the same force in agreement with the experimental observations in [8]. When the relaxation time and total viscosity increase in tandem through the polymer concentration, competing effects of elasticity and viscosity lead to a non-monotonic response of frequency on viscosity that again agrees well with experiments [8].

In [8], it was observed that although the frequency of the beat increased in viscoelastic fluids, the swimming speed decreased. The shape of the beat changed significantly in viscoelastic fluids, namely, the maximum of the flagellum curvature increased, but the bending at the basal end was reduced. In [39], we performed numerical simulations of swimmers based on the gaits from Qin *et al*. [8] to separate the effects of changes in gait and changes in fluid rheology on the swimming speed. This work showed that the reduction in speed resulted from both the change in the shape of the beat and the nonlinear growth of elastic stress around the flagella. The model analysed in this work does not predict shape changes in response to viscoelasticity. At low amplitude, the shape is determined by the eigenfunctions of the linearized operator, which do not depend on the fluid elasticity. Although the frequency response predicted from our analysis is consistent with Qin *et al*. [8], the reported shape changes in the flagella beat in response to fluid elasticity cannot be captured with the model analysed here. Capturing shape changes owing to fluid elasticity requires a more sophisticated model of the active forces from molecular motors.

The model we analysed did not include mechanical feedback on the driving force. There are many theories about how mechanical feedback on molecular motors leads to spatio-temporal coordination of motor activity to produce the flagellum beat, but it has also been shown that the motor coordination is not necessary for producing the beat [25]. Even if the mechanical feedback on motor activity is not responsible for coordination, motor regulation could play a role in modulating the flagellum beat. Our analysis captures the qualitative changes in the frequency observed in [8] in response to fluid elasticity, but the quantitative agreement between the model and data would likely require a more sophisticated model that includes dynamic motor activity. For example, our analysis predicts that in a Newtonian fluid, the frequency is inversely proportional to the viscosity (see also [25]), but as discussed in [8], the motor model from Camalet & Jülicher [19] predicts that the frequency is inversely proportional to the square root of the viscosity, which was a better fit to the data (see figure 8).

One approach to analysing how the shape and frequency of the flagellum beat are shaped by motor models that incorporate mechanical feedback is to examine time-periodic solutions of linearized equations [19,20,23,24]. This approach is equivalent to examining the solutions at the bifurcation point as we have done here. The form of linearized equations, and thus the eigenvalues of the corresponding operator, depends on the motor model, but the eigenvalues in a viscous fluid are related to those in a viscoelastic fluid by equation (3.6). This suggests that the effect of fluid elasticity on frequency discussed here may be a generic effect in models of flagella which incorporate feedback or regulation from molecular motors.

## Data Availability

Computer code is available from [39]. Electronic supplementary material is available online [40].
